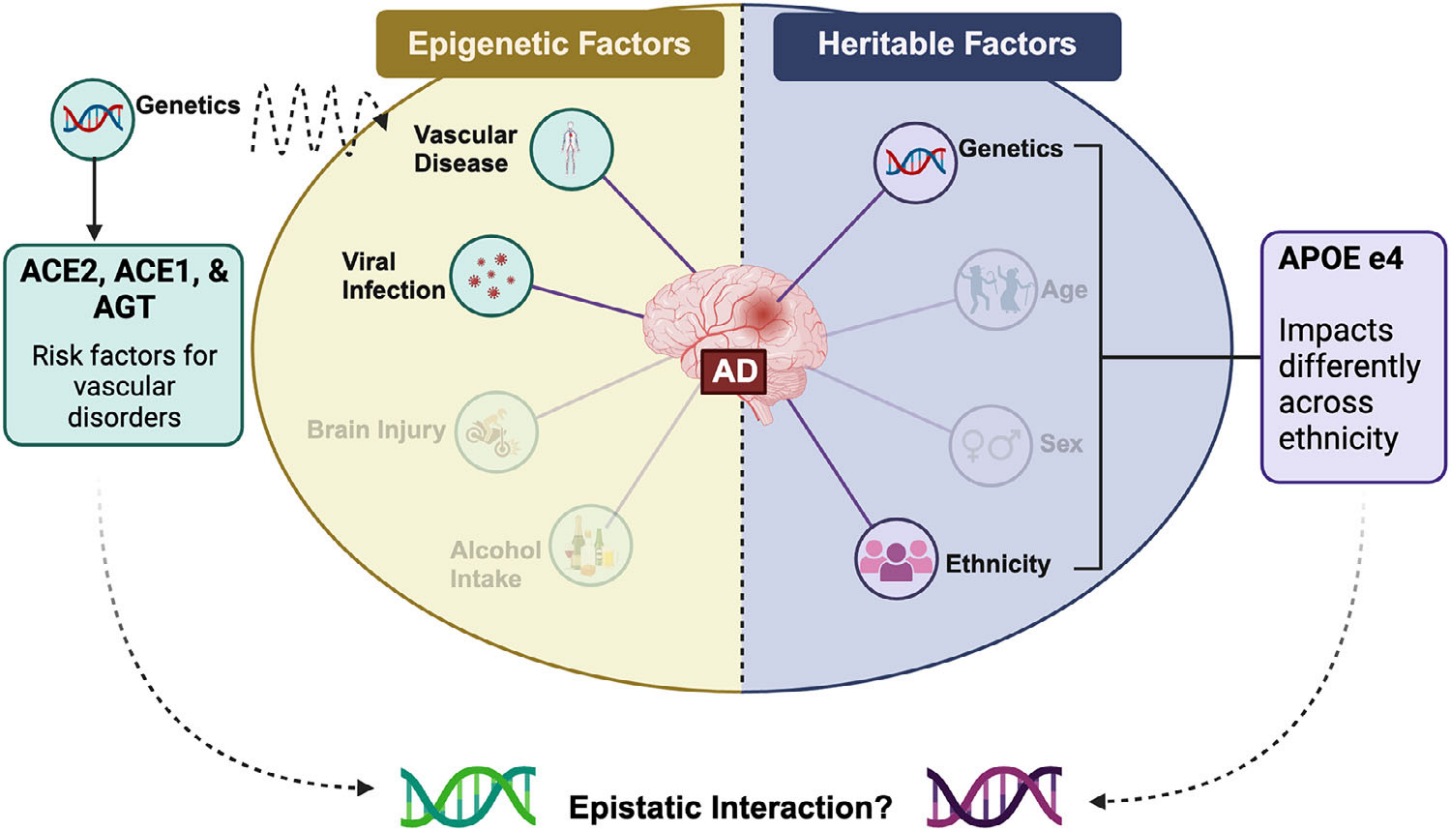# APOE epistasis as population specific risk factors for Alzheimer's Disease outcomes

**DOI:** 10.1002/alz70855_103802

**Published:** 2025-12-24

**Authors:** Amanda Elizabeth Tucker, Robert Barber, Nicole Phillips, Harlan P Jones

**Affiliations:** ^1^ University of North Texas Health Science Center, Fort Worth, TX, USA

## Abstract

**Background:**

Alzheimer's disease (**AD**) and AD related dementias (**ADRD**) have no single identifiable cause but it is understood that many risk factors may interplay to create AD sub‐phenotypes. The strongest genetic risk factor for development of AD/ADRD is the presence of the ε4 allele in the apolipoprotein E (**APOE**) gene, however the allele frequency of ε4 and its impact on cognitive decline can vary significantly across racial/ethnic groups. Vascular disorders are also strong risk factors for AD/ADRD. A multiethnic cohort study found that controlling for vascular diseases mediated the AD health disparity observed across racial/ethnic groups, highlighting the influence of vascular health on cognitive functions and neurodegeneration across these populations. Genetic variants in angiotensin converting enzymes (**ACE1** & **ACE2**) and angiotensinogen (**AGT**) are risk factors for vascular diseases due to the prominent role they play in the renin‐angiotensin aldosterone systems (**RAAS**) regulation of vasculature. Recent studies suggest a potential interaction between ACE2 & APOE proteins, making RAAS mediating genes plausible candidates for epistatic studies connecting vascular diseases and AD/ADRD risks. **We hypothesize that RAAS genetic loci have population specific SNPs associated with vascular disease and AD/ADRD, and that these contribute to group differences in AD risk and accelerated neurological dysfunction when found in epistasis with APOE**.

**Method:**

Genomic association analysis was conducted using the Healthy Aging Brain Study‐Health Disparities (**HABS‐HD**) dataset. RAAS gene variants implicated in vascular disorders within Mexican‐Americans, Black, and Non‐Hispanic White populations were identified, followed by epistasis, allele frequency, and genotype distribution analysis to determine if gene‐gene interactions differentially impact comorbidity‐based risk for AD/ADRD in high‐risk racial/ethnic populations.

**Result:**

We identified SNPs in ACE1 and AGT loci as candidates for translational studies on epistasis as a mechanism for differential AD outcomes. For each cohort, there were unique SNPs associated with each group and with different sets of phenotypes. We anticipate the same will occur in *in vitro studies*.

**Conclusion:**

This study begins elucidating disease comorbidity and gene‐gene interactions as a potential considerations for predicting AD risk, specifically a co‐occurance of RAAS gene SNPs with APOE, and supports future mechanistic studies on cellular phenotypes associated with epistatic effects.